# The Role of the N-Methyl-D-Aspartate Receptors in Social Behavior in Rodents

**DOI:** 10.3390/ijms20225599

**Published:** 2019-11-09

**Authors:** Iulia Zoicas, Johannes Kornhuber

**Affiliations:** Department of Psychiatry and Psychotherapy, University Hospital, Friedrich-Alexander-University Erlangen-Nuremberg, 91054 Erlangen, Germany; johannes.kornhuber@uk-erlangen.de

**Keywords:** NMDAR agonists, NMDAR antagonists, social investigation, ultrasonic vocalizations, social recognition, inter-male aggression, mating behavior, maternal care, maternal aggression

## Abstract

The appropriate display of social behaviors is essential for the well-being, reproductive success and survival of an individual. Deficits in social behavior are associated with impaired N-methyl-D-aspartate (NMDA) receptor-mediated neurotransmission. In this review, we describe recent studies using genetically modified mice and pharmacological approaches which link the impaired functioning of the NMDA receptors, especially of the receptor subunits GluN1, GluN2A and GluN2B, to abnormal social behavior. This abnormal social behavior is expressed as impaired social interaction and communication, deficits in social memory, deficits in sexual and maternal behavior, as well as abnormal or heightened aggression. We also describe the positive effects of pharmacological stimulation of the NMDA receptors on these social deficits. Indeed, pharmacological stimulation of the glycine-binding site either by direct stimulation or by elevating the synaptic glycine levels represents a promising strategy for the normalization of genetically-induced, pharmacologically-induced or innate deficits in social behavior. We emphasize on the importance of future studies investigating the role of subunit-selective NMDA receptor ligands on different types of social behavior to provide a better understanding of the underlying mechanisms, which might support the development of selective tools for the optimized treatment of disorders associated with social deficits.

## 1. Introduction

The majority of the excitatory neurotransmission in the mammalian central nervous system (CNS) is mediated by L-glutamate, which activates both presynaptic and postsynaptic ionotropic and metabotropic receptors (iGluRs and mGluRs). iGluRs are ligand-gated cation channels and can be divided into three classes: the α-amino-3-hydroxy-5-methyl-4-isoxasolepropionic acid (AMPA) receptors, kainate receptors, and N-methyl-D-aspartate (NMDA) receptors [1]. The NMDA receptors are named after the pharmacological agonist to whom they selectively respond and they function as ligand-gated channels. In addition to L-glutamate, NMDA receptors require the binding of a co-agonist, glycine or D-serine, to allow activation of the receptor, which then leads to the opening of a non-selective ion channel that allows the influx of Na^+^ and Ca^2+^ into the cell and the efflux of K^+^ out of the cell [2]. Because of their additional property of voltage-gating, the NMDA receptors function as coincidence-detectors. At resting membrane potentials, a hydrated Mg^2+^ binds to a site within the channel and inactivates the NMDA receptor. L-glutamate can only activate the receptor when the membrane is sufficiently depolarized to relieve this Mg^2+^ blockade. Therefore, the likelihood that L-glutamate leads to channel opening is dependent on the activation of AMPA receptors and membrane depolarization [3].

The NMDA receptors are heteromeric complexes formed of four constituent polypeptides, which arrange to form the ion-conductive pore [4,5,6,7]. Most NMDA receptors contain two obligatory GluN1 subunits (also called NR1), which create the ligand binding domain for glycine and D-serine and two GluN2 (also called NR2) subunits, which create the ligand binding domain for L-glutamate. The GluN2 subunits can be of four different types: GluN2A, GluN2B, GluN2C and GluN2D and the expression of these specific GluN2 subunits depends on the developmental age and brain region. As such, GluN2B and GluN2D subunits predominate in the fetal brain and generally decline throughout development, whereas the expression of GluN2A subunits increases after birth and predominates over GluN2B in the olfactory bulb, cortex, hippocampus and cerebellum by postnatal days 14–21 in rats and mice [8,9,10,11,12]. In the mature brain, GluN2C is highly expressed in the cerebellum, whereas GluN2D is highly expressed in subcortical regions and the brainstem. GluN1 and GluN2A are abundantly expressed in virtually all regions of the adult brain [8,13,14], including brain regions involved in social behavior, such as the olfactory bulbs, medial prefrontal cortex, amygdala, striatum, hippocampus, nucleus accumbens, septum and hypothalamus (reviewed in [15,16]). Mature NMDA receptors can be diheterotetrameric (i.e., GluN1/GluN2A or GluN1/GluN2B) and triheterotetrameric (i.e., GluN1/GluN2A/GluN2B) and their function is influenced by the diversity and the combination of their subunits (reviewed in [3,17]). Most cortical, amygdalar and hippocampal NMDA receptors are diheterotetrameric GluN1/GluN2A or GluN1/GluN2B receptors or triheterotetrameric GluN1/GluN2A/GluN2B receptors [13,18,19]. While the GluN2B-containing diheterotetrameric NMDA receptors have a preferential extra-synaptic localization and promote pro-apoptotic and excitotoxic processes, the GluN2A-containing NMDA receptors are predominantly synaptically-localized and may enhance the inhibitory drive of parvalbumin-expressing GABAergic neurons, while avoiding neurotoxic consequences associated with excessive stimulation of extra-synaptic GluN2B-containing receptors [4,19]. Although both excitatory and inhibitory neurons express high levels of GluN2A receptors, the synaptic GluR2A-containing NMDA receptors are present in a much higher density in inhibitory neurons [1,3]. It is estimated that GluN2A-containing NMDA receptors account for about 68% of synaptic currents and only about 27% of extra-synaptic currents in the mature forebrain, whereas the rest is accounted for by GluN2B-containing receptors [20]. It was also suggested that D-serine may be the preferred co-agonist in synaptically-located NMDA receptors, whereas glycine may be the preferred co-agonist in extra-synaptic NMDA receptors [21]. A regulatory GluN3 (also called NR3) subunit may also be present in the receptor complex, particularly during development [22].

The appropriate display of social behavior is critical for the well-being, reproductive success and survival of an individual as it allows for group living and successful interaction with other members of the species, obtaining food and avoiding predation. Social behavior includes all kind of behaviors that bring individuals together, i.e., social interaction and communication, reproductive and parental behavior, as well as all forms of aggressive behavior [23]. The importance of social behavior is also suggested by the high number of psychiatric disorders associated with social deficits, including social anxiety disorder [24], autism spectrum disorders [25], depression [26], schizophrenia [26], Alzheimer’s disease [26], alcohol use disorder [27] and fragile X syndrome [28], among others.

In this review we will describe the involvement of the NMDA receptor-mediated neurotransmission in regulating social behavior and summarize some of the strategies aimed at facilitating NMDA receptor function, which were shown to improve deficits in several aspects of social behavior in rodents. 

## 2. Role of the NMDA Receptors in Social Interaction

Social interactions are complex behaviors which require multiple cognitive processes and evaluation of social stimuli. A key aspect of social interaction is the motivation of an individual to initiate and respond to another individual. Social interaction is thought to produce a rewarding effect which reinforces and promotes approach behaviors [29,30]. The assessment of social interaction in rodents is based on their preference to spend time with another conspecific rather than remaining alone or to investigate social rather than non-social stimuli, i.e., objects, toys, etc. [31,32,33]. Rodents with impaired social behavior show decreased interaction or investigation of conspecifics or decreased time spent in a chamber containing an enclosed conspecific in tests such as the social interaction test, social preference test and the three-chambered social approach test (reviewed in [34]).

### 2.1. Genetic Models of Impaired NMDA Receptor Functioning; Effects on Social Interaction

Several studies using genetically modified mice demonstrated the importance of NMDA receptor-mediated neurotransmission in the regulation of social interaction in rodents (Table 1). As such, mice expressing only 5% of normal levels of the essential NMDA receptor GluN1 subunit (NR1(neo−/−) mice) show deficits in social interaction when housed with wild-type littermates, i.e., remain physically distant while wild-type littermates sleep piled in a nest [35], initiate and maintain less social interactions [35,36,37], avoid social interactions with littermates in the social interaction test [35] and spend less time in the social compartment of the three-chambered social approach test [37]. Similarly, knock-out mice with loss of GluN1 subunit in parvalbumin-containing GABAergic interneurons during development also show reduced social motivation and impaired social interaction [38,39]. Belforte et al. [40] have demonstrated deficits in social interaction in mice in which the GluN1 subunit was selectively eliminated in 40–50% of cortical and hippocampal GABAergic interneurons in early postnatal development, but not in the post-adolescent period, suggesting that early postnatal inhibition of the NMDA receptor activity in corticolimbic GABAergic interneurons contributes to the development of social deficits. However, Jacobs and Tsien [41] demonstrated that forebrain-specific GluN1-containing NMDA receptors modulate social motivation independent of neuronal development. As such, profound deficits in social motivation and social investigation were found in adult mice with inducible forebrain-specific deletion of GluN1 subunits prior to sociability testing [41]. A similarly impaired social behavior has been observed in Grin1^D481N^ mice, a genetic model of chronic and developmentally diminished NMDA receptor glycine site occupancy. These mice have a fivefold decrease in NMDA receptor glycine affinity due to a point mutation in the GluN1 glycine binding site [42] and show impaired social investigation in the three-chambered social approach test [43]. Impaired social investigation has also been described in serine racemase knock-out mice [44], which show an impaired NMDA receptor function due to a 90% reduction in cortical D-serine, an endogenous co-agonist acting on the NMDA receptor glycine-binding site, whose availability is dependent on the activity of the enzyme serine racemase [45].

### 2.2. Effects of the NMDA Receptor Antagonists on Social Interaction

Pharmacological studies also revealed social interaction deficits after administration of several types of NMDA receptor antagonists (Table 2). The NMDA receptor antagonists can be categorised in four classes: competitive antagonists bind to and block the glutamate binding site, glycine antagonists block the glycine binding site, non-competitive antagonists block the allosteric binding sites and un-competitive antagonists block the ion channel by binding to a site within it. Most of the studies used un-competitive antagonists, such as 5R,10S-(+)-5-methyl-10,11-dihydro-5H-dibenzo[a,d]cyclohepten-5,10-imine hydrogen maleate (MK-801, also known as dizocilpine), phencyclidine (PCP) and ketamine, to induce social interaction deficits and to mimic the negative symptoms of schizophrenia. As such, MK-801 decreased social interaction following both acute [46,47,48,49,50,51,52,53,54] and sub-chronic [55] treatment in adult rats and mice. Similarly, acute [56,57], sub-chronic [58,59,60,61] and chronic [62] administration of PCP decreased social interaction in adult rats and mice. Impairments in social interaction were also described in adult rats treated with PCP on postnatal days 7, 9 and 11 [63] and during adolescence on postnatal days 50 and 51 [64], suggesting that inhibition of NMDA receptor activity both during development and adulthood induces long-lasting changes in social behavior. Long-lasting social interaction deficits were also described by Qiao et al. [65] in mice during withdrawal from chronic PCP treatment (for a review see [66]). Similar to MK-801 and PCP, ketamine was shown to decrease social interaction following both acute [67,68,69,70] and sub-chronic [71,72,73] administration in rats and mice. 

Interestingly, studies using acute administration of GluN2A and GluN2B subtype-selective competitive antagonists revealed age-dependent differences in the social interaction test. As such, adolescent rats required higher doses of MK-801 and of the GluN2B-preferring antagonist ifenprodil to reduce social interactions than adults did, whereas an opposite age effect was observed after administration of {NVP-AAM o77; P-[[[(15)-1-(4-Bromophenyl)ethyl]amino](1,2,3,4-tetrahydro-2,3-dioxo-5-qyuinoxalinyl)methyl]phosphoric acid tetrasodium salt} (PEAQX), a GluN2A-preferring antagonist [4,49]. These results suggest that social interaction in adolescence is more sensitive to GluN2A-selective antagonism, whereas social interaction in adulthood is more sensitive to NMDA- and GluN2B-selective receptor antagonism and indicate that these differences may be related to different subunit expression patterns during development. As such, GluN2B subunits predominate in the fetal brain and generally decline throughout development, whereas the expression of GluN2A subunits increases after birth and predominates over GluN2B in the cortex, hippocampus and cerebellum by postnatal days 14-21 in rats and mice [8,9,10,11,12]. Interestingly, the NMDA receptor antagonists 1-amino-3,5-dimethyladamantane hydrochloride (memantine) reduced the social interaction deficits in mice prenatally treated with citalopram [74]. These effects of memantine, however, are not so surprising given that prenatal citalopram treatment increased GluN1 expression in the striatum [74]. Similarly, memantine reduced the social interaction deficits in IRSp53 knock-out mice [75] which show increased NMDA receptor function in the hippocampus [76], suggesting that deviations in NMDA receptor functioning in either direction (see also Section 2.3.) lead to deficits in social interaction and that correcting this deviation has beneficial effects.

### 2.3. Effects of the NMDA Receptor Agonists on Social Interaction

Studies using genetically modified mice and pharmacological approaches indicate that a decreased NMDA receptor function is associated with social interaction deficits (Tables 1 and 2). Since direct stimulation of the L-glutamate-binding site of the NMDA receptors can produce excitotoxic neuronal death [77,78], the enhancement of the NMDA receptor function by targeting the glycine-binding site may be more beneficial. There are two feasible ways of stimulating the glycine-binding site of the NMDA receptor, i.e., by direct stimulation of the glycine-binding site and by elevating the synaptic glycine levels. Agonists of the glycine-binding site of the NMDA receptors include glycine, D-serine, D-cycloserine, GLYX-13, vinyl glycine and a series of 3-acylamino-2-aminopropionic acid derivatives [79,80,81].

The positive effects of D-serine, a full agonist of the glycine-binding site of the NMDA receptors, have been demonstrated in inbred autistic Balb/c mice that show impaired social interaction [82,83,84,85] and heightened behavioral sensitivity to MK-801 [86,87,88] (Table 2). As such, acute administration of D-serine improved social investigation in Balb/c mice and increased the time spent in the social compartment of the three-chambered social approach test [89]. D-serine also improved the social investigation deficits of Grin1^D481N^ mice, which show decreased NMDA receptor glycine affinity due to a point mutation in the GluN1 glycine binding site [42] and impaired social investigation [46]. Interestingly, long-term increases in D-serine through a genetic inactivation of its catabolic enzyme D-amino acid oxidase (DAO) was shown to reverse the social interaction deficits of Grin1^D481N^ mice, without altering social investigation in wild-type mice [90], suggesting that overstimulation of the glycine-binding site of the NMDA receptors does not negatively affect social interaction. Furthermore, Nagai et al. [91] have shown that D-serine ameliorates social interaction deficits by potentiating the NMDA receptor activity. As such, mice treated with polyriboinosinic-polyribocytidilic acid (polyI:C; a synthetic analog that elicits viral-like immune responses) during early postnatal development show social interaction deficits which could be improved by D-serine treatment in adulthood, an effect which was antagonized by pretreatment with the un-competitive NMDA-receptor antagonist MK-801 [91].

D-cycloserine, a partial agonist of the glycine-binding site of NMDA receptors was also effective in improving the social interaction deficits observed in autistic Balb/c mice [85,92,93,94], in autistic BTBR T+tf/J mice [95] and in autistic Shank2 knock-out mice, which show diminished expression of the Shank2 postsynaptic scaffolding protein and decreased function of the GluN2A- and GluN2B-containing NMDA receptors in the hippocampus [96]. Acute administration of D-cycloserine also normalized the autistic-like alterations caused by prenatal exposure to the anticonvulsant drug valproic acid in rats [97,98] and improved the social interaction deficits induced by postnatal PCP treatment in mice, without altering social investigation in control mice [99]. 

Another way to stimulate the glycine-binding site of the NMDA receptors is by elevating the synaptic glycine levels. This can be achieved by inhibiting the glycine transporter 1 (GlyT1), which removes glycine from the synaptic cleft and is closely associated with the NMDA receptor [100]. Competitive GlyT1 inhibitors such as sarcosine (N-methylglycine), N[3-(4′-flurophenyl)-3-(4′-phenylphenoxy) propyl]sarcosine (NFPS, a sarcosine analogue) and 3-chloro-N-{(S)-[3-(1-ethyl-1H-pyrazol-4-yl)phenyl][(2S)-piperidin-2-yl]methyl}-4-(trifluoromethyl)pyridine-2-carboxamide (TASP0315003) are, therefore, expected to increase the NMDA receptor function by indirectly activating the glycine-binding site. Increasing the NMDA receptor function by both acute and sub-chronic administration of TASP0315003 was shown to reverse PCP-induced deficits in social interaction, without altering social investigation in control mice [101]. Another selective high-affinity competitive GlyT1 inhibitor, 2,4-dichloro-N-((4-(cyclopropylmethyl)-1-(ethyl sulfonyl)piperidin-4-yl)methyl)benzamide (VU0410120), improved the social interaction deficits of autistic Balb/c mice [102], further demonstrating the efficacy of GlyT1 inhibitors.

## 3. Role of the NMDA Receptors in Social Communication

Many vertebrates use species-specific vocalizations to communicate information regarding identity, group status, mood, environmental conditions (presence of predators or location of food) and to facilitate mother-offspring interactions. Rats emit three types of ultrasonic vocalizations (USV) depending on age, environmental conditions and affective state. As such, 50 kHz USV are emitted under non-aversive conditions, such as juvenile play [103,104], sexual behavior [105] and during manual tactile stimulation (“tickling”) by experimenters [106,107]. These 50 kHz USV are thought to reflect a positive affective state. Rats can also emit 22 kHz USV in aversive situations, such as exposure to predators [108], inescapable pain induced by foot shocks [109], in response to startling noises [110] and during social defeat and inter-male aggression [111]. These 22 kHz USV are thought to reflect a negative affective state. Unlike rats, mice produce different types of USV, which are not indicators of positive or negative affective states but likely function to facilitate or inhibit social interactions [112]. As such, male mice emit USV mainly during mating behaviors (in the presence of females or their pheromones), i.e., 70 kHz “pre-mating” USV, which help to coordinate the mating process, and 40 kHz “mating” USV [113,114]. Female mice, on the other hand, emit USV when alone, searching for pups or in the presence of other females [115]. Rat and mouse pups emit so called “distress-calls” when they are separated from their mother. These USV have frequencies of 40 kHz (rat pups) and 70–100 kHz (mouse pups) and are thought to reflect a negative affective state [112].

### 3.1. Genetic Models of Impaired NMDA Receptor Functioning; Effects on Social Communication

Altered social communication has been described in genetically modified mice that show deficits in social behavior (Table 1). As such, male NR1(neo-/-) mice that express only 5% of normal levels of the essential NMDA receptor GluN1 subunit show deficits in social interaction [35,36,37] and fewer “pre-mating” 70 kHz USV during mating behavior [116]. Similarly, knock-out mice with loss of GluN1 subunit in parvalbumin-containing GABAergic interneurons during development, which show reduced social motivation and impaired social interaction [38,39], also show reduced “pre-mating” 70 kHz USV [38]. Reduced social communication through USV was also described in autistic Shank2 knock-out mice, which show decreased function of the GluN2A- and GluN2B-containing NMDA receptors in the hippocampus and impaired social interaction [96]. When allowed to interact with a receptive female, male Shank2 knock-out mice emitted less frequently USV and had a longer latency to make the first call [96]. Serine racemase knock-out mice, which show impaired social investigation and an impaired NMDA receptor function due to a 90% reduction in cortical D-serine [45], also emit fewer USV in the presence of a conspecific [44].

### 3.2. Effects of the NMDA Receptor Antagonists and Agonists on Social Communication

In rats, diminished social interaction is typically associated with a lower amount of 50 kHz USV [117]. Accordingly, pharmacological manipulations that impair social interaction in rats (see Section 2.2) were shown to reduce 50 kHz USV (Table 2). Boulay et al. [118] observed a decrease in 50 kHz USV in rats exposed to human tickling after an acute PCP injection. Similarly, 2-ethylamino-2-thiophen-2-yl-cyclohexan-1-one (tiletamine, an un-competitive NMDA receptor antagonist) and ketamine reduced social interaction and accompanying 50 kHz USV in rats after acute administration [70]. Altered social communication was also described in rats prenatally exposed to the anticonvulsant drug valproic acid, which is known to induce an autistic-like phenotype [97,98]. Interestingly, acute administration of D-cycloserine, a partial agonist of the glycine-binding site of the NMDA receptors, reversed the valproic acid-induced deficits in social interaction and reduced the changes in USV [97]. Moskal et al. [119] described a rat line selectively bred for high and low rates of rough-and-tumble play-induced 50 kHz USV. Low line rats show lower social interaction, lower rates of play-induced pro-social 50 kHz USV and an increased proportion of monotonous USV compared with randomly bred rats. While 50 kHz USV were associated with pro-social positive affective states [120], monotonous USV were associated with communication deficits [121,122,123]. GLYX-13, a partial agonist of the glycine-binding site of the NMDA receptors, increased rates of play-induced pro-social USV and decreased the proportion of monotonous USV, indicating that GLYX-13 can reverse the social communication deficit in low line rats by activating the NMDA receptors [80,119].

Reduced social communication was also described in rat and mice pups treated with several types of NMDA receptor antagonists. As such, separation-induced USV in rat pups were reduced by competitive antagonists (which block the glutamate-binding site), such as D,L-amino-phosphonovaleric acid (AP5), 2-amino-7-phosphonoheptanoic acid (AP7), 3-((+/-)-2-carboxypiperazin-4-yl)-propyl-1-phosphonic acid (CPP) and MDL 100,453 [124,125,126], by un-competitive antagonists (which block the ion channel by binding to a site within it), such as MK-801 [125] and by glycine antagonists (which block the glycine-binding site), such as 5,7-dichlorokynurenic acid (5,7-DCKA) [125]. Administration of NMDA was effective in both increasing USV by almost 50% and in reversing the effects of AP7 [124]. Interestingly, Takahashi et al. [127] reported that the separation-induced USV in mouse pups are differently affected by high versus low affinity un-competitive antagonists. As such, the high affinity antagonist MK-801 dose-dependently reduced USV, whereas the low affinity NMDA receptor antagonists memantine and neramexane showed biphasic effects and enhanced USV at low to moderate doses. How MK-801 and memantine/neramexane differently modulate USV is yet unclear and needs further investigation, but might relate to different receptor binding kinetics and affinities of these antagonists. High affinity MK-801 has very slow kinetics, whereas low affinity memantine is voltage-dependent and has quick blocking and unblocking kinetics [128,129], which might be due to their diverse binding affinity to different NMDA receptor subtypes [130,131].

## 4. Role of the NMDA Receptors in Social Memory

Individual recognition in socially living species is essential for the development of normal social relationships and for the establishment of hierarchies that function to limit aggressive interactions and to allow group living [132,133]. In most mammals, social recognition is achieved through information encoded by olfactory and pheromonal signals [134]. The assessment of social memory in rodents is based on their tendency to investigate unfamiliar conspecifics more intensely than familiar ones. Intact social memory is, therefore, indicated by a reduced investigation of previously encountered conspecifics [135] in the social recognition test or in the social discrimination test.

### 4.1. Genetic Models of Impaired NMDA Receptor Functioning; Effects on Social Memory

Several studies using genetically modified mice demonstrated the importance of NMDA receptor-mediated neurotransmission in the regulation of social memory in rodents (Table 1). As such, GluN2D knock-out mice with deficient expression of the GluN2D subunit of the NMDA receptors exhibited a decreased preference for social novelty, despite normal olfactory function and social interaction [136]. These GluN2D knock-out mice also showed a reduction in the complexity of dendritic trees in the accessory olfactory bulb, suggesting a deficit in pheromone-processing pathway activation which modulates social recognition memory [136]. By using forebrain-specific GluN2A overexpressing mice and forebrain-specific GluN2B overexpressing mice, Jacobs et al. [137] could demonstrate that postnatal overexpression of the GluN2A subunit of the NMDA receptors impairs social memory, while overexpression of the GluN2B subunit enhances social memory, i.e., prolongs recognition of juveniles and females of the same strain. Furthermore, forebrain-specific GluN2B overexpressing mice showed prolonged recognition of mice of a different strain and rodents of another species [138]. Deficits in social recognition memory were also described in mice in which the GluN1 subunit was selectively eliminated in 40%–50% of cortical and hippocampal GABAergic interneurons during postnatal development, but not during adulthood, suggesting that early postnatal inhibition of the NMDA receptor activity in corticolimbic GABAergic interneurons contributes to the development of social memory deficits [33]. However, Jacobs and Tsien [41] demonstrated that forebrain-specific GluN1-containing NMDA receptors modulate social motivation and social recognition independent of neuronal development. As such, an impaired social memory was observed in adult mice with inducible forebrain-specific deletion of GluN1 subunits prior to behavioral testing [41]. Interestingly, Chiang et al. [139] showed that deletion of the GluN1 subunit gene in the ventral, but not in the dorsal, CA3 pyramidal cells leads to deficits in social memory, while mice lacking the same gene in the DG granule cells showed intact social memory. These results suggest that ventral hippocampal CA3 pyramidal cell plasticity and transmission contribute to the encoding of social stimuli [139].

### 4.2. Effects of the NMDA Receptor Antagonists and Agonists on Social Memory

Pharmacological studies in rats and mice also revealed deficits in social memory after administration of NMDA receptor antagonists, such as MK-801 [101,140,141,142,143], PCP [63,144,145] and ketamine [146] (Table 2). Conversely, enhancement of the NMDA receptor function by targeting either the glutamate-binding site with NMDA [147] or the glycine-binding site was shown to enhance social recognition memory in rodents. As such, D-serine, a full agonist of the glycine-binding site of the NMDA receptors, was shown to prolong social memory in rats [140] and to improve the MK-801-induced deficits in social memory [143]. Pyrroloquinoline quinone (PQQ), a powerful neuroprotectant that specifically binds to the glycine-binding site of the brain NMDA receptors, also improved the MK-801-induced deficits in social memory and even accentuated the effects of D-serine when administered in combination [143]. Inhibition of glycine transporter 1 with the GlyT1-inhibitors N[3-(4′-flurophenyl)-3-(4′-phenylphenoxy) propyl]sarcosine (NFPS) and 3-chloro-N-{(S)-[3-(1-ethyl-1H-pyrazol-4-yl)phenyl][(2S)-piperidin-2-yl]methyl}-4-(trifluoromethyl)pyridine-2-carboxamide (TASP0315003), which increase the NMDA receptor function by indirectly activating the glycine-binding site, was shown to enhance social recognition in treatment-naïve rats and to prevent the MK-801-induced deficits in social memory [101,140]. Similarly, SSR-504734 and SSR130800, other GlyT1-inhibitors, improved the social recognition deficits induced by neonatal PCP treatment in rats [63,144]. SSR130800 was shown to reversibly block glycine uptake in mouse brain cortical homogenates, to increase extracellular levels of glycine in the rat prefrontal cortex and to potentiate NMDA-mediated excitatory postsynaptic currents in rat hippocampal slices [144].

## 5. Role of the NMDA Receptors in Inter-Male Aggression

The occurrence of inter-male aggression in rodents is usually related to territorial defensive behaviors or to the establishment and maintenance of the social status within a group [148]. The aggressive behavior of rodents can be assessed in a resident–intruder paradigm, where an unfamiliar male (intruder) is placed in the home cage of the experimental rodent (resident), which defends its territory and shows aggressive behavior towards the intruder [149,150]. Higher levels of aggression might be obtained when residents are co-housed with females before the test.

Several studies using genetically modified mice and pharmacological approaches investigated the role of NMDA receptor-mediated neurotransmission in the regulation of inter-male aggression in rodents (Tables 1 and 2). As such, mice expressing only 5% of normal levels of the essential NMDA receptor GluN1 subunit (NR1(neo-/-) mice) show less aggressive behaviors in a social interaction paradigm [37], reduced social investigation towards a male intruder mouse and reduced inter-male aggression in a resident-intruder paradigm [35,36], suggesting that the GluN1-containing NMDA receptors promote territorial aggressive behavior. The involvement of the GluN2-containing NMDA receptors in aggressive behavior seems to be more complex as it necessitates a fine-tuned balance of GluN2B expression in the brain. As such, mice lacking ST8 alpha-*N*-acetyl-neuraminide alpha-2,8-sialyltransferase 2, an enzyme with important function in neuroplasticity [151], show decreased NMDA receptor currents and a decreased expression of GluN2B, but not of GluN1 and GluN2A, in the lateral amygdala [152]. These St8sia2 knock-out mice show an abnormal aggressive behavior, characterized by an increased number of bites in vulnerable body parts, i.e., throat and belly, and an increased number of bites while the male intruder is already submissive. St8sia2 knock-out mice also show high levels of aggressiveness in a neutral arena against juvenile and female mice, a situation which usually does not trigger aggressive responses in wild-type mice [152]. D-cycloserine, a partial agonist of the glycine-binding site of the NMDA receptors, reversibly increased the NMDA receptor currents in both wild-type and St8sia2 knock-out mice and normalized the abnormal aggressive behavior in St8sia2 knock-out mice [152]. D-cycloserine also increased affiliative behavior in wild-type mice, i.e., increased social investigation and decreased aggression in resident mice [153]. Interestingly, both high- and low-affinity un-competitive antagonists were shown to decrease aggressive behavior in mice. As such, the high-affinity antagonists MK-801 and ketamine decreased bite frequency in resident mice [154] and reduced the number of attacks in socially-isolated mice [155]. However, although social isolation was shown to increase inter-male aggressive behavior in rats and mice [156,157,158], it increased GluN2A and GluN2B expression in the hippocampus [159], suggesting that the decrease in the NMDA receptor currents induced by MK-801 and ketamine normalizes inter-male aggressive behavior. Similarly, the low-affinity NMDA receptor antagonists memantine and 1-amino-1,3,3,5,5-pentamethyl-cyclohexan hydrochloride (MRZ 2/579) reduced morphine withdrawal-facilitated aggression in mice [160], without affecting inter-male aggressive behavior in morphine-naïve mice [160,161]. Similarly to social isolation, morphine withdrawal was shown to increase inter-male aggressive behavior [162,163,164,165] and to increase the GluN2A and GluN2B expression in the frontal cortex and the GluN2B expression in the striatum [166]. It is, therefore, not surprising that the NMDA receptor antagonists reduced aggressive behavior in individuals with increased GluN2A and GluN2B expression in the brain. These results suggest that deviations in GluN2B- and possibly GluN2A-containing NMDA receptor function in either direction leads to abnormal inter-male aggressive behavior and that correcting this deviation has beneficial effects.

## 6. Role of the NMDA Receptors in Sexual Behavior

Male sexual behavior consists of a pattern of genital and somato-motor responses which are induced and maintained by pheromonal, hormonal and neural signals. It includes copulation and pre-copulatory behaviors that allow males to detect a female and facilitate a receptive response [167]. The copulatory behavior consists of mounts, intromissions and ejaculations and can be assessed by exposing males to receptive females [167]. Parameters such as mount latency and intromission latency are indicative of sexual motivation, whereas the latency to ejaculation, the number of mounts and intromissions before ejaculation and the time from ejaculation to the next intromission (i.e., post-ejaculation interval) are measures of sexual performance [167].

Most of the studies investigated the role of the GluN1-containing NMDA receptors in sexual behavior (Tables 1 and 2). As such, NR1(neo-/-) mice, which express only 5% of normal levels of the essential NMDA receptor GluN1 subunit and have a normal function of reproductive organs, did not copulate with receptive wild-type females and failed to produce litters even after extended breeding [35]. This abnormal sexual behavior seems to be facilitated by the impaired sociability of male NR1(neo-/-) mice [35,36,37], as treatment with clozapine, which increased social approach and reduced social withdrawal in mice [168], also increased copulatory behavior up to 30% of normal levels seen in wild-type males [35]. A decreased breeding efficiency was also reported in mice in which the GluN1 subunit was selectively eliminated in 40%–50% of cortical and hippocampal GABAergic interneurons in early postnatal development [40]. These mice showed a decreased copulatory behavior, which was not the result of reproductive organ dysfunctions, as pairs that successfully bred produced normal-sized litters. These two studies suggest that impaired GluN1-containing NMDA receptor-mediated neurotransmission induces deficits in copulatory behavior which might be facilitated by the accompanying deficits in social interaction.

Several studies have shown that both systemic and intra-medial preoptic area (MPOA) injections of the un-competitive NMDA receptor antagonist MK-801 impaired sexual behavior in male and female rats [169,170,171]. As such, systemic administration of MK-801 impaired copulatory behavior in sexually naïve and sexually experienced male rats [171] and impaired female sexual behavior in rats [172]. Injections of MK-801 into the MPOA, a brain region essential for the expression of male sexual behavior [173], impaired copulatory behavior in sexually naïve and sexually experienced rats [169,170], i.e., decreased the numbers of mounts, intromissions and ejaculations and increased the latencies for these measures. Dominguez et al. [169] demonstrated that almost all MPOA neurons that were activated, i.e., expressed c-Fos, following mating contained the GluN1 subunit of the NMDA receptors and that mating increased phosphorylation (activation) of GluN1 in the MPOA. They could also show that blockade of the NMDA receptors by MK-801 in the MPOA decreased mating-induced c-Fos expression and phosphorylation of NMDA receptors and impaired male sexual behavior [169]. These results demonstrate that mating activates the GluN1-containing NMDA receptors in the MPOA and that this activation is important for the expression of male sexual behavior.

## 7. Role of the NMDA Receptors in Maternal Behavior

Maternal behavior consists of a group of behaviors exhibited by the mother that promotes the survival of the offspring and includes maternal care and maternal aggression [174]. Maternal care refers to the nurturing of the offspring and includes behaviors such as nest building, retrieval of pups and crouching over pups, whereas maternal aggression has the function of protecting the pups and the nest [174,175].

There are only a few studies investigating the role of NMDA receptor-mediated neurotransmission in maternal behavior (Table 1 and Table 2). As such, impaired pup retrieval, as an indicator of impaired maternal care, was described in autistic Shank2 knock-out mice, which show impaired sociability and a decreased GluN2A- and GluN2B-containing NMDA receptor function in the hippocampus [96]. Severe deficits in maternal behavior were also observed in Hbegf knock-out mice [176], which have a genetic deletion of the heparin-binding epidermal growth factor-like growth factor (HB-EGF). These mice show reduced expression of hippocampal GluN1/GluN2-NMDA receptor subunits and reduced expression of PSD-95, a scaffold protein involved in the NMDA receptor aggregation in the CNS [177]. The impaired maternal behavior of Hbegf knock-out mice was reflected by a lack of nest building, impaired pup retrieval and lack of nursing (lactation behavior), which resulted in a delayed pup growth and decreased pup survival [176].

Muroi and Ishii [178] demonstrated that administration of AP5, a competitive NMDA receptor antagonist, in the dorsal raphe nucleus, a brain region that regulates both maternal care and maternal aggression [179,180], inhibited maternal aggression, but not maternal care. In contrast, injections of NMDA into the dorsal raphe nucleus impaired maternal care, i.e., pup retrieval, pup licking and crouching over the pups, suggesting that glutamatergic inputs to the dorsal raphe nucleus regulate the preferential display of maternal care over maternal aggression [178]. Interestingly, the un-competitive NMDA receptor antagonist MK-801 blocked experienced-based facilitation of maternal care [181]. As such, when unexperienced mothers were administered MK-801 before the first contact with the pups, they showed no maternal care, i.e., couching over the pups and licking the pups, when tested 10 days later. However, when MK-801 was administered following one hour of contact with the pups, the females showed unaltered maternal care at a later time-point, suggesting that the NMDA receptor-mediated neurotransmission is required for the long-term experienced-based facilitation of maternal care [181].

## 8. Conclusions

Recent discoveries from genetically modified mice and pharmacological studies using NMDA receptor antagonists link the impaired function of the NMDA receptors to abnormal social behavior, such as impaired social interaction and communication, deficits in social memory, deficits in sexual and maternal behavior, as well as abnormal or heightened aggression. Pharmacological stimulation of the glycine-binding site, either by direct stimulation or by elevating the synaptic glycine levels, represents a promising strategy for the normalization of genetically-induced, pharmacologically-induced or innate deficits in social behavior. This accumulating evidence and the identification of new subunit-selective NMDA receptor modulators make further studies investigating their potentially beneficial role worthwhile. Apart from providing a better understanding of the neural mechanisms involved and regulated by different NMDA receptor subunits, these investigations might stimulate the development of selective tools for the improved treatment of psychiatric disorders associated with social deficits.

## Figures and Tables

**Table 1:** Expression of NMDA receptors in animal models with impaired social behavior.

Species	NMDA Receptor Functioning	Behavioral Deficit	References
NR1(neo-/-) mice	Expression of GluN1 only to 5% of normal levels	Impaired social interaction Impaired social communication Reduced inter-male aggression Impaired sexual behavior	[35,36,37] [116] [35,36,37] [35]
PV-selective NR1 knock-out mice	Loss of GluN1 subunit in parvalbumin-containing GABAergic interneurons during development	Impaired social interaction Impaired social communication	[38,39] [38]
*Ppp1r2-cre^+/−^; NR1^loxP/loxP^ mice*	Selective elimination of GluN1 in 40%–50% of cortical and hippocampal GABAergic interneurons in early postnatal development, but not in the post-adolescent period	Impaired social interaction Impaired social memory Impaired sexual behavior	[40]
iFB knock-out mice	Inducible forebrain-specific deletion of GluN1 subunits	Impaired social interaction Impaired social memory	[41]
CA3 NR1 knock-out mice	Deletion of the GluN1 subunit gene in the ventral, but not the dorsal, CA3 pyramidal cells	Impaired social memory	[139]
DG NR1 knock-out mice	Deletion of the GluN1 subunit gene in DG granule cells	Normal social memory	[139]
GluN2B^2A(CTD)^ mice	Increased postnatal forebrain expression of the GluN2A subunit	Impaired social memory	[137]
GluN2A^2B(CTD)^ mice	Increased postnatal forebrain expression of the GluN2B subunit	Enhanced social memory	[137,138]
GluN2D knock-out mice	Deficient expression of the GluN2D subunit	Normal social interaction Impaired social memory	[136]
Grin1^D481N^ mice	Chronic and developmentally diminished NMDA receptor glycine site occupancy (fivefold decrease in NMDA receptor glycine affinity)	Impaired social interaction	[42,43]
Serine racemase knock-out mice	Impaired NMDA receptor function due to a 90% reduction in cortical D-serine	Impaired social interaction Impaired social communication	[44]
Hbegf knock-out mice	Reduced expression of hippocampal GluN1/GluN2-NMDA receptor subunits Reduced expression of PSD-95, a scaffold protein involved in the NMDA receptor aggregation in the CNS	Impaired maternal behavior	[176,177]
Shank2 knock-out mice	Decreased function of the GluN2A- and GluN2B-containing NMDA receptors in the hippocampus	Impaired social interaction Impaired social communication Impaired maternal behavior	[96]
St8sia2 knock-out mice	Decreased NMDA receptor currents and decreased expression of GluN2B, but not of GluN1 and GluN2A, in the lateral amygdala	Abnormal (psychopathological) aggressive behavior	[152]
Socially-isolated mice and rats	Increased GluN2A and GluN2B expression in the hippocampus Increased GluN2A expression in the prefrontal cortex	Impaired social interaction Altered social communication Impaired social memory Increased inter-male aggression Impaired sexual behavior	[182,183] [184] [185] [156,157,158,159] [186,187,188]
Mice during morphine withdrawal	Increased GluN2A and GluN2B expression in the frontal cortex Increased GluN2B expression in the striatum	Increased inter-male aggression	[162,163,164,165,166]

NMDA, N-methyl-D-aspartate; NR1, GluN1 subunit; PV, parvalbumin; GABA, gamma aminobutyric acid; iFB, inducible forebrain-specific expression; CA3, cornu ammonis 3; DG, dentate gyrus; NR2, GluN2 subunit.

**Table 2:** Effects of the NMDA receptor agonists and antagonists on social behavior in rodents.

Action	Drug	Species	Behavioral Change	References
**Effects on social interaction**				
Un-competitive NMDA receptor antagonists	MK-801 PCP Ketamine	Mice and rats	Decreased social interaction	[46,47,48,49,50,51,52,53,54,55] [56,57,58,59,60,61,62,63,64,65,66] [67,68,69,70,71,72,73]
NMDA receptor antagonist	Memantine	IRSp53 knock-out mice Prenatally citalopram-treated mice	Reduced social interaction deficits	[74,75]
GluN2A-preferring antagonist	PEAQX	Rats	Decreased social interaction	[4,49]
GluN2B-preferring antagonist	Ifenprodil	Rats	Decreased social interaction	[49]
Full agonist of the glycine-binding site	D-serine	Autistic Balb/c mice Grin1^D481N^ mice Postnatally polyI:C-treated mice	Reduced social interaction deficits	[89] [90] [91]
Partial agonist of the glycine-binding site	D-cycloserine	Autistic Balb/c mice Autistic BTBR T+tf/J mice Autistic Shank2 knock-out mice Prenatally VPA-treated mice Postnatally PCP-treated mice	Reduced social interaction deficits	[85,92,93,94] [95] [96] [98] [99]
GlyT1 inhibitor	TASP0315003 VU0410120	Postnatally PCP-treated mice Autistic Balb/c mice	Reduced social interaction deficits	[101] [102]
**Social communication (USV)**				
Un-competitive NMDA receptor antagonists	PCP Tiletamine Ketamine	Rats	Reduced 50 kHz USV	[118] [70] [70]
Un-competitive NMDA receptor antagonist	MK-801	Rat pups	Reduced separation-induced USV	[125,127]
Competitive NMDA receptor antagonists	AP5 AP7 CPP MDL 100,453	Rat pups	Reduced separation-induced USV	[124,126]
NMDA receptor antagonists	Memantine Neramexane	Rat pups	Increased separation-induced USV at low to moderate doses and reduced separation-induced USV at high doses	[127]
Glycine antagonist	5,7-DCKA	Rat pups	Reduced separation-induced USV	[125]
NMDA receptor agonist	NMDA	Rat pups	Increased separation-induced USV	[124]
Partial agonist of the glycine-binding site	D-cycloserine GLYX-13	Prenatally VPA-treated rats Rats selectively bred for low rates of play-induced 50 kHz USV	Reduced deficits in social communication Reversed deficits in social communication	[97] [80,119]
**Social memory**				
Un-competitive NMDA receptor antagonists	MK-801 PCP Ketamine	Rats and mice	Impaired social memory	[101,140,141,142,143] [63,144,145,146]
Full agonists of the glycine-binding site	D-serine PQQ	Rats MK-801-treated rats	Enhanced social memory Improved social memory deficits	[140] [143]
GlyT1 inhibitors	NFPS TASP0315003 SSR-504734 SSR130800	Rats MK-801-treated rats Neonatally PCP-treated rats	Enhanced social memory Improved social memory deficits	[140] [101] [63] [144]
**Inter-male aggression**				
Un-competitive NMDA receptor antagonists	MK-801 Ketamine	Socially-isolated mice	Decreased inter-male aggression	[155] [154]
NMDA receptor antagonists	Memantine MRZ 2/579	Naïve and morphine-treated mice	Reduced morphine withdrawal-facilitated aggression, but did not affect inter-male aggression in naïve mice	[160,161] [160,161]
Partial agonist of the glycine-binding site	D-cycloserine	Mice St8sia2 knock-out mice	Decreased inter-male aggression Normalized the abnormal aggressive behavior	[153] [152]
**Sexual behavior**				
Un-competitive NMDA receptor antagonist	MK-801	Male and female rats	Impaired sexual behavior	[169,170,171,172]
**Maternal behavior**				
Un-competitive NMDA receptor antagonist	MK-801	Rats	Blocked experienced-based facilitation of maternal care	[181]
Competitive NMDA receptor antagonist	AP5	Mice	Impaired maternal aggression, but not maternal care	[178]
NMDA receptor agonist	NMDA	Mice	Impaired maternal care	[178]

NMDA, N-methyl-D-aspartate; MK-801, 5R,10S-(+)-5-methyl-10,11-dihydro-5H-dibenzo[a,d]cyclohepten-5,10-imine hydrogen maleate; MK-801, dizocilpine, 5R,10S-(+)-5-methyl-10,11-dihydro-5H-dibenzo[a,d]cyclohepten-5,10-imine hydrogen maleate; PCP, phencyclidine; memantine, 1-amino-3,5-dimethyladamantane hydrochloride; PEAQX, {NVP-AAM o77; P-[[[(15)-1-(4-Bromophenyl)ethyl]amino](1,2,3,4-tetrahydro-2,3-dioxo-5-qyuinoxalinyl)methyl]phosphoric acid tetrasodium salt}; polyI:C, polyriboinosinic-polyribocytidilic acid; VPA, valproic acid; GlyT1, glycine transporter 1; TASP0315003, 3-chloro-N-{(S)-[3-(1-ethyl-1H-pyrazol-4-yl)phenyl][(2S)-piperidin-2-yl]methyl}-4-(trifluoromethyl)pyridine-2-carboxamide; VU0410120, 2,4-dichloro-N-((4-(cyclopropylmethyl)-1-(ethyl sulfonyl)piperidin-4-yl)methyl)benzamide; USV, ultrasonic vocalizations; tiletamine, 2-ethylamino-2-thiophen-2-yl-cyclohexan-1-one; AP5, D,L-amino-phosphonovaleric acid; AP7, 2-amino-7-phosphonoheptanoic acid; CPP, 3-((+/-)-2-carboxypiperazin-4-yl)-propyl-1-phosphonic acid; memantine, 1-amino-3,5-dimethyladamantane hydrochloride; 5,7-DCKA, 5,7-dichlorokynurenic acid; PQQ, pyrroloquinoline quinone; NFPS, N[3-(4′-flurophenyl)-3-(4′-phenylphenoxy) propyl]sarcosine; MRZ 2/579, 1-amino-1,3,3,5,5-pentamethyl-cyclohexan hydrochloride.

## References

[1] Traynelis S.F., Wollmuth L.P., McBain C.J., Menniti F.S., Vance K.M., Ogden K.K., Hansen K.B., Yuan H., Myers S.J., Dingledine R. (2010). Glutamate receptor ion channels: Structure, regulation, and function. Pharmacol. Rev..

[2] Danysz W., Parsons C.G., Parsons C. (1998). GlycineB recognition site of NMDA receptors and its antagonists. Amino Acids.

[3] Hansen K.B., Yi F., Perszyk R.E., Furukawa H., Wollmuth L.P., Gibb A.J., Traynelis S.F. (2018). Structure, function, and allosteric modulation of NMDA receptors. J. Gen. Physiol..

[4] Green T.L., Burket J.A., Deutsch S.I. (2016). Age-dependent effects on social interaction of NMDA GluN2A receptor subtype-selective antagonism. Brain Res. Bull..

[5] Hackos D.H., Lupardus P.J., Grand T., Chen Y., Wang T.-M., Reynen P., Gustafson A., Wallweber H.J., Volgraf M., Sellers B.D. (2016). Positive Allosteric Modulators of GluN2A-Containing NMDARs with Distinct Modes of Action and Impacts on Circuit Function. Neuron.

[6] Hackos D.H., Hanson J.E. (2017). Diverse modes of NMDA receptor positive allosteric modulation: Mechanisms and consequences. Neuropharmacol..

[7] Xiang Z., Conn P.J. (2016). Novel PAMs Targeting NMDAR GluN2A Subunit. Neuron.

[8] Monyer H., Burnashev N., Laurie D.J., Sakmann B., Seeburg P.H. (1994). Developmental and regional expression in the rat brain and functional properties of four NMDA receptors. Neuron.

[9] Sheng M., Cummings J., Roldan L.A., Jan Y.N., Jan L.Y. (1994). Changing subunit composition of heteromeric NMDA receptors during development of rat cortex. Nat..

[10] Wenzel A., Fritschy J.M., Mohler H., Benke D. (1997). NMDA receptor heterogeneity during postnatal development of the rat brain: Differential expression of the NR2A, NR2B, and NR2C subunit proteins. J. Neurochem..

[11] Liu X.-B., Murray K.D., Jones E.G. (2004). Switching of NMDA Receptor 2A and 2B Subunits at Thalamic and Cortical Synapses during Early Postnatal Development. J. Neurosci..

[12] Zhang X.-M., Luo J.-H. (2013). GluN2A versus GluN2B: Twins, but quite different. Neurosci. Bull..

[13] Paoletti P., Bellone C., Zhou Q. (2013). NMDA receptor subunit diversity: Impact on receptor properties, synaptic plasticity and disease. Nat. Rev. Neurosci..

[14] Sanz-Clemente A., Nicoll R.A., Roche K.W. (2013). Diversity in NMDA receptor composition: Many regulators, many consequences. Neuroscientist.

[15] Ko J. (2017). Neuroanatomical Substrates of Rodent Social Behavior: The Medial Prefrontal Cortex and Its Projection Patterns. Front. Neural Circuits.

[16] Chen P., Hong W. (2018). Neural Circuit Mechanisms of Social Behavior. Neuron.

[17] Cull-Candy S., Brickley S., Farrant M. (2001). NMDA receptor subunits: Diversity, development and disease. Curr. Opin. Neurobiol..

[18] Conti J. (1997). Localization of NMDA receptors in the cerebral cortex: A schematic overview. Braz. J. Med. Biol. Res..

[19] De Armentia M.L., Sah P. (2003). Development and Subunit Composition of Synaptic NMDA Receptors in the Amygdala: NR2B Synapses in the Adult Central Amygdala. J. Neurosci..

[20] Zhou X., Ding Q., Chen Z., Yun H., Wang H. (2013). Involvement of the GluN2A and GluN2B Subunits in Synaptic and Extrasynaptic N-methyl-d-aspartate Receptor Function and Neuronal Excitotoxicity*. J. Boil. Chem..

[21] Vizi E., Kisfali M., Lorincz T. (2013). Role of nonsynaptic GluN2B-containing NMDA receptors in excitotoxicity: Evidence that fluoxetine selectively inhibits these receptors and may have neuroprotective effects. Brain Res. Bull..

[22] Das S., Sasaki Y.F., Rothe T., Premkumar L.S., Takasu M., Crandall J.E., Dikkes P., Conner D.A., Rayudu P.V., Cheung W. (1998). Increased NMDA current and spine density in mice lacking the NMDA receptor subunit NR3A. Nat..

[23] Alexander R.D. (1974). The evolution of social behavior. Annu. Rev. Ecol. Syst..

[24] Dryman M.T., Heimberg R.G. (2018). Emotion regulation in social anxiety and depression: A systematic review of expressive suppression and cognitive reappraisal. Clin. Psychol. Rev..

[25] Frye R.E. (2018). Social Skills Deficits in Autism Spectrum Disorder: Potential Biological Origins and Progress in Developing Therapeutic Agents. CNS Drugs.

[26] Porcelli S., Van Der Wee N., van der Werff S., Aghajani M., Glennon J.C., van Heukelum S., Mogavero F., Lobo A., Olivera F.J., Lobo E. (2018). Social brain, social dysfunction and social withdrawal. Neurosci. Biobehav. Rev..

[27] Oliveira L.M., Bermudez M.B., Macedo M.J.D.A., Passos I.C. (2018). Comorbid social anxiety disorder in patients with alcohol use disorder: A systematic review. J. Psychiatr. Res..

[28] Hagerman R.J., Protic D., Rajaratnam A., Salcedo-Arellano M.J., Aydin E.Y., Schneider A. (2018). Fragile X-Associated Neuropsychiatric Disorders (FXAND). Front. Psychol..

[29] Krach S., Paulus F.M., Bodden M., Kircher T. (2010). The Rewarding Nature of Social Interactions. Front. Behav. Neurosci..

[30] Trezza V., Campolongo P., Vanderschuren L.J. (2011). Evaluating the rewarding nature of social interactions in laboratory animals. Dev. Cogn. Neurosci..

[31] File S.E., Hyde J. (1978). CAN SOCIAL INTERACTION BE USED TO MEASURE ANXIETY?. Br. J. Pharmacol..

[32] Berton O. (2006). Essential Role of BDNF in the Mesolimbic Dopamine Pathway in Social Defeat Stress. Science.

[33] Lukas M., Toth I., O Reber S., A Slattery D., Veenema A.H., Neumann I.D. (2011). The Neuropeptide Oxytocin Facilitates Pro-Social Behavior and Prevents Social Avoidance in Rats and Mice. Neuropsychopharmacology.

[34] Toth I., Neumann I.D. (2013). Animal models of social avoidance and social fear. Cell and Tissue Research.

[35] Mohn A.R., Gainetdinov R.R., Caron M.G., Koller B.H. (1999). Mice with reduced NMDA receptor expression display behaviors related to schizophrenia. Cell.

[36] E Duncan G., Moy S.S., Pérez A., Eddy D.M., Zinzow W.M., A Lieberman J., Snouwaert J.N., Koller B.H. (2004). Deficits in sensorimotor gating and tests of social behavior in a genetic model of reduced NMDA receptor function. Behav. Brain Res..

[37] Halene T.B., Ehrlichman R.S., Liang Y., Christian E.P., Jonak G.J., Gur T.L., Blendy J.A., Dow H.C., Brodkin E.S., Schneider F. (2009). Assessment of NMDA receptor NR1 subunit hypofunction in mice as a model for schizophrenia. Genes, Brain Behav..

[38] Saunders J.A., Tatard-Leitman V.M., Suh J., Billingslea E.N., Roberts T.P., Siegel S.J. (2013). Knockout of NMDA receptors in parvalbumin interneurons recreates autism-like phenotypes. Autism Res..

[39] Billingslea E.N., Tatard-Leitman V.M., Anguiano J., Jutzeler C.R., Suh J., A Saunders J., Morita S., E Featherstone R., I Ortinski P., Gandal M.J. (2014). Parvalbumin Cell Ablation of NMDA-R1 Causes Increased Resting Network Excitability with Associated Social and Self-Care Deficits. Neuropsychopharmacol..

[40] Belforte J.E., Zsiros V., Sklar E.R., Jiang Z., Yu G., Li Y., Quinlan E.M., Nakazawa K. (2010). Postnatal NMDA receptor ablation in corticolimbic interneurons confers schizophrenia-like phenotypes. Nat. Neurosci..

[41] Jacobs S., Tsien J.Z. (2017). Adult forebrain NMDA receptors gate social motivation and social memory. Neurobiol. Learn. Mem..

[42] Kew J.N.C., Koester A., Moreau J.-L., Jenck F., Ouagazzal A.-M., Mutel V., Richards J.G., Trube G., Fischer G., Montkowski A. (2000). Functional Consequences of Reduction in NMDA Receptor Glycine Affinity in Mice Carrying Targeted Point Mutations in the Glycine Binding Site. J. Neurosci..

[43] Labrie V., Lipina T., Roder J.C. (2008). Mice with reduced NMDA receptor glycine affinity model some of the negative and cognitive symptoms of schizophrenia. Psychopharmacology.

[44] Matveeva T.M., Pisansky M.T., Young A., Miller R.F., Gewirtz J.C. (2019). Sociality deficits in serine racemase knockout mice. Brain Behav..

[45] Basu A.C., Tsai G.E., Ma C.L., Ehmsen J.T., Mustafa A.K., Han L., Jiang Z.I., Benneyworth M.A., Froimowitz M.P., Lange N. (2009). Targeted disruption of serine racemase affects glutamatergic neurotransmission and behavior. Mol. Psychiatry..

[46] Rung J.P., Carlsson A., Markinhuhta K.R., Carlsson M.L. (2005). (+)-MK-801 induced social withdrawal in rats; a model for negative symptoms of schizophrenia. Prog. Neuro-Psychopharmacology Boil. Psychiatry.

[47] Satow A., Suzuki G., Maehara S., Hikichi H., Murai T., Murai T., Kawagoe-Takaki H., Hata M., Ito S., Ozaki S. (2009). Unique Antipsychotic Activities of the Selective Metabotropic Glutamate Receptor 1 Allosteric Antagonist 2-Cyclopropyl-5-[1-(2-fluoro-3-pyridinyl)-5-methyl-1H-1,2,3-triazol-4-yl]-2,3-dihydro-1H-isoindol-1-one. J. Pharmacol. Exp. Ther..

[48] Sławińska A., Wierońska J.M., Stachowicz K., Marciniak M., Lasoń-Tyburkiewicz M., Gruca P., Papp M., Kusek M., Tokarski K., Doller D. (2013). The antipsychotic-like effects of positive allosteric modulators of metabotropic glutamate mGluR4 receptors in rodents. Br. J. Pharmacol..

[49] Morales M., Spear L.P. (2014). The effects of an acute challenge with the NMDA receptor antagonists, MK-801, PEAQX, and ifenprodil, on social inhibition in adolescent and adult male rats. Psychopharmacology (Berl)..

[50] Wierońska J.M., Kłeczek N., Woźniak M., Gruca P., Łasoń-Tyburkiewicz M., Papp M., Brański P., Burnat G., Pilc A. (2015). mGlu₅-GABAB interplay in animal models of positive, negative and cognitive symptoms of schizophrenia. Neurochem. Int..

[51] Wierońska J.M., Sławińska A., Łasoń-Tyburkiewicz M., Gruca P., Papp M., Zorn S.H., Doller D., Kłeczek N., Noworyta-Sokołowska K., Gołembiowska K. (2015). The antipsychotic-like effects in rodents of the positive allosteric modulator Lu AF21934 involve 5-HT1A receptor signaling: Mechanistic studies. Psychopharmacology (Berl)..

[52] Woźniak M., Acher F., Marciniak M., Lasoń-Tyburkiewicz M., Gruca P., Papp M., Pilc A., Wierońska J.M. (2016). Involvement of GABAB receptor signaling in antipsychotic-like action of the novel orthosteric agonist of the mGlu4 receptor, LSP4-2022. Curr. Neuropharmacol..

[53] Cieślik P., Woźniak M., Rook J.M., Tantawy M.N., Conn P.J., Acher F., Tokarski K., Kusek M., Pilc A., Wierońska J.M. (2018). Mutual activation of glutamatergic mGlu4 and muscarinic M4 receptors reverses schizophrenia-related changes in rodents. Psychopharmacology.

[54] Cieślik P., Woźniak M., Kaczorowska K., Brański P., Burnat G., Chocyk A., Bobula B., Gruca P., Litwa E., Pałucha-Poniewiera A. (2018). Negative Allosteric Modulators of mGlu7 Receptor as Putative Antipsychotic Drugs. Front. Mol. Neurosci..

[55] Matsuoka T., Sumiyoshi T., Tanaka K., Tsunoda M., Uehara T., Itoh H., Kurachi M. (2005). NC-1900, an arginine–vasopressin analogue, ameliorates social behavior deficits and hyperlocomotion in MK-801-treated rats: Therapeutic implications for schizophrenia. Brain Res..

[56] Sams-Dodd F. (1998). A test of the predictive validity of animal models of schizophrenia based on phencyclidine and D-amphetamine. Neuropsychopharmacol..

[57] Slot L.A.B., Kleven M.S., Newman-Tancredi A. (2005). Effects of novel antipsychotics with mixed D2 antagonist/5-HT1A agonist properties on PCP-induced social interaction deficits in the rat. Neuropharmacol..

[58] Lee P.R., Brady D.L., A Shapiro R., Dorsa D.M., I Koenig J. (2005). Social Interaction Deficits Caused by Chronic Phencyclidine Administration are Reversed by Oxytocin. Neuropsychopharmacol..

[59] Snigdha S., Neill J. (2008). Efficacy of antipsychotics to reverse phencyclidine-induced social interaction deficits in female rats—A preliminary investigation. Behav. Brain Res..

[60] Snigdha S., Neill J.C. (2008). Improvement of phencyclidine-induced social behaviour deficits in rats: Involvement of 5-HT1A receptors. Behav. Brain Res..

[61] Audet M.-C., Goulet S., Doré F.Y. (2009). Impaired social motivation and increased aggression in rats subchronically exposed to phencyclidine. Physiol. Behav..

[62] Peters S.M., Tuffnell J.A., Pinter I.J., Van Der Harst J.E., Spruijt B.M. (2017). Short- and long-term behavioral analysis of social interaction, ultrasonic vocalizations and social motivation in a chronic phencyclidine model. Behav. Brain Res..

[63] Harich S., Gross G., Bespalov A. (2007). Stimulation of the metabotropic glutamate 2/3 receptor attenuates social novelty discrimination deficits induced by neonatal phencyclidine treatment. Psychopharmacology.

[64] White I.M., Minamoto T., Odell J.R., Mayhorn J., White W. (2009). Brief exposure to methamphetamine (METH) and phencyclidine (PCP) during late development leads to long-term learning deficits in rats. Brain Res..

[65] Qiao H., Noda Y., Kamei H., Nagai T., Furukawa H., Miura H., Kayukawa Y., Ohta T., Nabeshima T. (2001). Clozapine, but not haloperidol, reverses social behavior deficit in mice during withdrawal from chronic phencyclidine treatment. NeuroReport.

[66] Neill J.C., Barnes S., Cook S., Grayson B., Idris N.F., McLean S.L., Snigdha S., Rajagopal L., Harte M.K. (2010). Animal models of cognitive dysfunction and negative symptoms of schizophrenia: Focus on NMDA receptor antagonism. Pharmacol. Ther..

[67] Silvestre J.S., Nadal R., Pallarès M., Ferré N. (1997). Acute effects of ketamine in the holeboard, the elevated-plus maze, and the social interaction test in Wistar rats. Depression Anxiety.

[68] Nikiforuk A., Hołuj M., Kos T., Popik P. (2016). The effects of a 5-HT 5A receptor antagonist in a ketamine-based rat model of cognitive dysfunction and the negative symptoms of schizophrenia. Neuropharmacol..

[69] Zugno A.I., Canever L., Heylmann A.S., Wessler P.G., Steckert A., Mastella G.A., De Oliveira M.B., Damázio L.S., Pacheco F.D., Calixto O.P. (2016). Effect of folic acid on oxidative stress and behavioral changes in the animal model of schizophrenia induced by ketamine. J. Psychiatr. Res..

[70] Popik P., Hołuj M., Kos T., Nowak G., Librowski T., Sałat K. (2017). Comparison of the Psychopharmacological Effects of Tiletamine and Ketamine in Rodents. Neurotox. Res..

[71] Becker A., Peters B., Schroeder H., Mann T., Huether G., Grecksch G. (2003). Ketamine-induced changes in rat behaviour: A possible animal model of schizophrenia. Prog. Neuro-Psychopharmacology Boil. Psychiatry.

[72] Koros E., Rosenbrock H., Birk G., Weiss C., Sams-Dodd F. (2007). The selective mGlu5 receptor antagonist MTEP, similar to NMDA receptor antagonists, induces social isolation in rats. Neuropsychopharmacology.

[73] Zoupa E., Gravanis A., Pitsikas N. (2019). The novel dehydroepiandrosterone (DHEA) derivative BNN27 counteracts behavioural deficits induced by the NMDA receptor antagonist ketamine in rats. Neuropharmacol..

[74] Zahra A., Jiang J., Chen Y., Long C., Yang L. (2018). Memantine rescues prenatal citalopram exposure-induced striatal and social abnormalities in mice. Exp. Neurol..

[75] Chung W., Choi S.Y., Lee E., Park H., Kang J., Park H., Choi Y., Lee D., Park S.-G., Kim R. (2015). Social deficits in IRSp53 mutant mice improved by NMDAR and mGluR5 suppression. Nat. Neurosci..

[76] Kim M.H., Choi J., Yang J., Chung W., Kim J.H., Paik S.K., Kim K., Han S., Won H., Bae Y.S. (2009). Enhanced NMDA Receptor-Mediated Synaptic Transmission, Enhanced Long-Term Potentiation, and Impaired Learning and Memory in Mice Lacking IRSp53. J. Neuroscience.

[77] Choi D., Maulucci-Gedde M., Kriegstein A. (1987). Glutamate neurotoxicity in cortical cell culture. J. Neurosci..

[78] Choi D.W. (1992). Excitotoxic cell death. J. Neurobiol..

[79] Urwyler S., Floersheim P., Roy B.L., Koller M. (2009). Drug Design, in Vitro Pharmacology, and Structure−Activity Relationships of 3-Acylamino-2-aminopropionic Acid Derivatives, a Novel Class of Partial Agonists at the Glycine Site on theN-Methyl-d-aspartate (NMDA) Receptor Complex. J. Med. Chem..

[80] Santini A.C., Pierantoni G.M., Gerlini R., Iorio R., Olabinjo Y., Giovane A., Di Domenico M., Sogos C. (2014). Glix 13, a New Drug Acting on Glutamatergic Pathways in Children and Animal Models of Autism Spectrum Disorders. BioMed Res. Int..

[81] Maolanon A.R., Risgaard R., Wang S.-Y., Snoep Y., Papangelis A., Yi F., Holley D., Barslund A.F., Svenstrup N., Hansen K.B. (2017). Subtype-Specific Agonists for NMDA Receptor Glycine Binding Sites. ACS Chem. Neurosci..

[82] Sankoorikal G.M.V., Kaercher K.A., Boon C.J., Lee J.K., Brodkin E.S. (2006). A Mouse Model System for Genetic Analysis of Sociability: C57BL/6J Versus BALB/cJ Inbred Mouse Strains. Boil. Psychiatry.

[83] Brodkin E. (2007). BALB/c mice: Low sociability and other phenotypes that may be relevant to autism. Behav. Brain Res..

[84] Moy S.S., Nadler J.J., Young N.B., Perez A., Holloway L.P., Barbaro R.P., Barbaro J.R., Wilson L.M., Threadgill D.W., Lauder J.M. (2007). Mouse behavioral tasks relevant to autism: Phenotypes of 10 inbred strains. Behav. Brain Res..

[85] Deutsch S.I., Burket J.A., Jacome L.F., Cannon W.R., Herndon A.L. (2011). d-Cycloserine improves the impaired sociability of the Balb/c mouse. Brain Res. Bull..

[86] Deutsch S.I., Rosse R.B., Paul S.M., Riggs R.L., Mastropaolo J. (1997). Inbred mouse strains differ in sensitivity to “popping” behavior elicited by MK-Pharmacol. Biochem. Behav..

[87] Deutsch S.I., Mastropaolo J., Powell D.G., Rosse R.B., Bachus S.E. (1998). Inbred mouse strains differ in their sensitivity to an antiseizure effect of MK-801. Clin. Neuropharmacol..

[88] Burket J.A., Cannon W.R., Jacome L.F., Deutsch S.I. (2010). MK-801, a noncompetitive NMDA receptor antagonist, elicits circling behavior in the genetically inbred Balb/c mouse strain. Brain Res. Bull..

[89] Jacome L.F., Burket J.A., Herndon A.L., Cannon W.R., Deutsch S.I. (2011). D-serine improves dimensions of the sociability deficit of the genetically-inbred Balb/c mouse strain. Brain Res. Bull..

[90] Labrie V., Wang W., Barger S.W., Baker G.B., Roder J.C. (2010). Genetic loss of D-amino acid oxidase activity reverses schizophrenia-like phenotypes in mice. Genes Brain Behav..

[91] Nagai T., Yu J., Kitahara Y., Nabeshima T., Yamada K. (2012). D-Serine Ameliorates Neonatal PolyI:C Treatment^|^ndash;Induced Emotional and Cognitive Impairments in Adult Mice. J. Pharmacol. Sci..

[92] Jacome L.F., Burket J.A., Herndon A.L., Deutsch S.I. (2011). d-Cycloserine enhances social exploration in the Balb/c mouse. Brain Res. Bull..

[93] Deutsch S.I., Pepe G.J., Burket J.A., Winebarger E.E., Herndon A.L., Benson A.D. (2012). d-cycloserine improves sociability and spontaneous stereotypic behaviors in 4-week old mice. Brain Res..

[94] Benson A.D., Burket J.A., Deutsch S.I. (2013). Balb/c mice treated with d-cycloserine arouse increased social interest in conspecifics. Brain Res. Bull..

[95] Burket J.A., Benson A.D., Tang A.H., Deutsch S.I. (2013). D-Cycloserine improves sociability in the BTBR T+ Itpr3tf/J mouse model of autism spectrum disorders with altered Ras/Raf/ERK1/2 signaling. Brain Res. Bull..

[96] Won H., Lee H.-R., Gee H.Y., Mah W., Kim J.-I., Lee J., Ha S., Chung C., Jung E.S., Cho Y.S. (2012). Autistic-like social behaviour in Shank2-mutant mice improved by restoring NMDA receptor function. Nature.

[97] Wellmann K.A., Varlinskaya E.I., Mooney S.M. (2014). D-Cycloserine ameliorates social alterations that result from prenatal exposure to valproic acid. Brain Res. Bull..

[98] Wu H.F., Chen P.S., Hsu Y.T., Lee C.W., Wang T.F., Chen Y.J., Lin H.C. (2018). D-cycloserine ameliorates autism-like deficits by removing GluA2-containing AMPA receptors in a valproic acid-induced rat model. Mol. Neurobiol..

[99] Nakatani-Pawlak A., Yamaguchi K., Tatsumi Y., Mizoguchi H., Yoneda Y. (2009). Neonatal phencyclidine treatment in mice induces behavioral, histological and neurochemical abnormalities in adulthood. Boil. Pharm. Bull..

[100] Smith K.E., Borden L.A., Hartig P.R., Branchek T., Weinshank R.L. (1992). Cloning and expression of a glycine transporter reveal colocalization with NMDA receptors. Neuron.

[101] Chaki S., Shimazaki T., Karasawa J.-I., Aoki T., Kaku A., Iijima M., Kambe D., Yamamoto S., Kawakita Y., Shibata T. (2015). Efficacy of a glycine transporter 1 inhibitor TASP0315003 in animal models of cognitive dysfunction and negative symptoms of schizophrenia. Psychopharmacology.

[102] Burket J.A., Benson A.D., Green T.L., Rook J.M., Lindsley C.W., Conn P.J., Deutsch S.I. (2015). Effects of VU0410120, a novel GlyT1 inhibitor, on measures of sociability, cognition and stereotypic behaviors in a mouse model of autism. Prog. Neuro-Psychopharmacology Boil. Psychiatry.

[103] Panksepp J. (1981). The ontogeny of play in rats. Devel. Psychobiol..

[104] Knutson B., Burgdorf J., Panksepp J. (1998). Anticipation of play elicits high-frequency ultrasonic vocalizations in young rats. J. Comp. Psychol..

[105] Barfield R.J., Geyer L.A., Wolf L.L., Hainsworth F.R., Stiles F.G. (1972). Sexual Behavior: Ultrasonic Postejaculatory Song of the Male Rat. Science.

[106] Brudzynski S.M., Ociepa D. (1992). Ultrasonic vocalization of laboratory rats in response to handling and touch. Physiol. Behav..

[107] Panksepp J., Burgdorf J. (2000). 50-kHz chirping (laughter?) in response to conditioned and unconditioned tickle-induced reward in rats: Effects of social housing and genetic variables. Behav. Brain Res..

[108] Blanchard R., Blanchard D., Agullana R., Weiss S.M. (1991). Twenty-two kHz alarm cries to presentation of a predator, by laboratory rats living in visible burrow systems. Physiol. Behav..

[109] Borta A., Wöhr M., Schwarting R. (2006). Rat ultrasonic vocalization in aversively motivated situations and the role of individual differences in anxiety-related behavior. Behav. Brain Res..

[110] Kaltwasser M.T. (1991). Acoustic startle induced ultrasonic vocalization in the rat: A novel animal model of anxiety?. Behav. Brain Res..

[111] Vivian J.A., Miczek K.A. (1993). Morphine attenuates ultrasonic vocalization during agonistic encounters with male rats. Psychopharmacology.

[112] Portfors C.V. (2007). Types and functions of ultrasonic vocalizations in laboratory rats and mice. J. Am. Assoc. Lab. Anim. Sci..

[113] Gourbal B.E.F., Barthelemy M., Petit G., Gabrion C. (2004). Spectrographic analysis of the ultrasonic vocalisations of adult male and female BALB/c mice. Naturwissenschaften.

[114] Ricceri L., Moles A., Crawley J. (2007). Behavioral phenotyping of mouse models of neurodevelopmental disorders: Relevant social behavior patterns across the life span. Behav. Brain Res..

[115] Ehret G. (2005). Infant Rodent Ultrasounds ? A Gate to the Understanding of Sound Communication. Behav. Genet..

[116] Gandal M.J., Anderson R.L., Billingslea E.N., Carlson G.C., Roberts T.P., Siegel S.J. (2012). Mice with reduced NMDA receptor expression: More consistent with autism than schizophrenia?. Genes, Brain Behav..

[117] Lukas M., Wöhr M. (2015). Endogenous vasopressin, innate anxiety, and the emission of pro-social 50-kHz ultrasonic vocalizations during social play behavior in juvenile rats. Psychoneuroendocrinology.

[118] Boulay D., Ho-Van S., Bergis O., Avenet P., Griebel G. (2013). Phencyclidine decreases tickling-648 induced 50-kHz ultrasound vocalizations in juvenile rats: A putative model of the negative symptoms of schizophrenia. Behav. Pharmacol..

[119] Moskal J.R., Burgdorf J., Kroes R.A., Brudzynski S.M., Panksepp J. (2011). A novel NMDA receptor glycine-site partial agonist, GLYX-13, has therapeutic potential for the treatment of autism. Neurosci. Biobehav. Rev..

[120] Burgdorf J., Kroes R.A., Weiss C., Oh M.M., Disterhoft J.F., Brudzynski S.M., Panksepp J., Moskal J.R. (2011). Positive emotional learning is regulated in the medial prefrontal cortex by GluN2B-containing NMDA receptors. Neurosci..

[121] Panksepp J. (1992). Oxytocin Effects on Emotional Processes: Separation Distress, Social Bonding, and Relationships to Psychiatric Disorders. Ann. New York Acad. Sci..

[122] Ciucci M.R., Ma S.T., Kane J.R., Ahrens A.M., Schallert T. (2008). Limb use and complex ultrasonic vocalization in a rat model of Parkinson’s disease: Deficit-targeted training. Park. Relat. Disord..

[123] Ciucci M.R., Ahrens A.M., Ma S.T., Kane J.R., Windham E.B., Woodlee M.T., Schallert T. (2009). Reduction of dopamine synaptic activity: Degradation of 50-kHz ultrasonic vocalization in rats. Behav. Neurosci..

[124] Winslow J.T., Insel T.R., Trullas R., Skolnick P. (1990). Rat pup isolation calls are reduced by functional antagonists of the NMDA receptor complex. Eur. J. Pharmacol..

[125] Kehne J.H., McCloskey T.C., Baron B.M., Chi E.M., Harrison B.L., Whitten J.P., Palfreyman M.G. (1991). NMDA receptor complex antagonists have potential anxiolytic effects as measured with separation-induced ultrasonic vocalizations. Eur. J. Pharmacol..

[126] Podhorna J., E Brown R. (2000). Interactions between N-methyl-D-aspartate and nitric oxide in the modulation of ultrasonic vocalizations of infant rats. Eur. J. Pharmacol..

[127] Takahashi A., Yap J.J., Bohager D.Z., Faccidomo S., Clayton T., Cook J.M., Miczek K.A. (2009). Glutamatergic and GABAergic modulations of ultrasonic vocalizations during maternal separation distress in mouse pups. Psychopharmacology (Berl)..

[128] Parsons C., Quack G., Bresink I., Baran L., Przegaliński E., Kostowski W., Krzascik P., Hartmann S., Danysz W., Parsons C. (1995). Comparison of the potency, kinetics and voltage-dependency of a series of uncompetitive NMDA receptor antagonists in vitro with anticonvulsive and motor impairment activity in vivo. Neuropharmacol..

[129] Parsons C.G., Stöffler A., Danysz W., Parsons C. (2007). Memantine: A NMDA receptor antagonist that improves memory by restoration of homeostasis in the glutamatergic system - too little activation is bad, too much is even worse. Neuropharmacol..

[130] Bresink I., Danysz W., Parsons C., Mutschler E., Parsons C. (1995). Different binding affinities of NMDA receptor channel blockers in various brain regions—Indication of NMDA receptor heterogeneity. Neuropharmacol..

[131] Kornhuber J., Bormann J., Retz W., Hübers M., Riederer P. (1989). Memantine displaces [3H]MK-801 at therapeutic concentrations in postmortem human frontal cortex. Eur. J. Pharmacol..

[132] Halpin Z.T. (1986). Individual Odors among Mammals: Origins and Functions. Adv. Study Behav..

[133] Hurst J.L., Fang J., Barnard C. (1994). The role of substrate odours in maintaining social tolerance between male house mice, Mus musculus domesticus: Relatedness, incidental kinship effects and the establishment of social status. Anim. Behav..

[134] Carr W.J., Yee L., Gable D., Marasco E. (1976). Olfactory recognition of conspecifics by domestic Norway rats. J. Comp. Physiol. Psychol..

[135] Thor D.H., Holloway W.R. (1981). Persistence of social investigatory behavior in the male rat: Evidence for long-term memory of initial copulatory experience. Learn. Behav..

[136] Yamamoto H., Kamegaya E., Hagino Y., Takamatsu Y., Sawada W., Matsuzawa M., Ide S., Yamamoto T., Mishina M., Ikeda K. (2017). Loss of GluN2D subunit results in social recognition deficit, social stress, 5-HT2C receptor dysfunction, and anhedonia in mice. Neuropharmacol..

[137] Jacobs S., Wei W., Wang D., Tsien J.Z. (2015). Importance of the GluN2B carboxy-terminal domain for enhancement of social memories. Learn. Mem..

[138] Jacobs S.A., Tsien J.Z. (2012). Genetic Overexpression of NR2B Subunit Enhances Social Recognition Memory for Different Strains and Species. PLOS ONE.

[139] Chiang M.-C., Huang A.J., Wintzer M.E., Ohshima T., McHugh T.J. (2018). A role for CA3 in social recognition memory. Behav. Brain Res..

[140] Shimazaki T., Kaku A., Chaki S. (2010). d-Serine and a glycine transporter-1 inhibitor enhance social memory in rats. Psychopharmacol.

[141] Hikichi H., Kaku A., Karasawa J.-I., Chaki S. (2013). Stimulation of metabotropic glutamate (mGlu) 2 receptor and blockade of mGlu1 receptor improve social memory impairment elicited by MK-801 in rats. J. Pharmacol. Sci..

[142] Hikichi H., Hiyoshi T., Marumo T., Tomishima Y., Kaku A., Iida I., Urabe H., Tamita T., Yasuhara A., Karasawa J.-I. (2015). Antipsychotic profiles of TASP0443294, a novel and orally active positive allosteric modulator of metabotropic glutamate 2 receptor. J. Pharmacol. Sci..

[143] Zhou X., Liu D., Zhang R., Peng Y., Qin X., Mao S. (2016). Modulation of glycine sites enhances social memory in rats using PQQ combined with d-serine. Behav. Brain Res..

[144] Boulay D., Pichat P., Dargazanli G., Estennebouhtou G., Terranova J., Rogacki N., Stemmelin J., Coste A., Lanneau C., Desvignes C. (2008). Characterization of SSR103800, a selective inhibitor of the glycine transporter-1 in models predictive of therapeutic activity in schizophrenia☆. Pharmacol. Biochem. Behav..

[145] Clifton N.E., Morisot N., Girardon S., Millan M.J., Loiseau F. (2013). Enhancement of social novelty discrimination by positive allosteric modulators at metabotropic glutamate 5 receptors: Adolescent administration prevents adult-onset deficits induced by neonatal treatment with phencyclidine. Psychopharmacology.

[146] Gao X.M., Elmer G.I., Adams-Huet B., Tamminga C.A. (2009). Social memory in mice: Disruption with an NMDA antagonist and attenuation with antipsychotic drugs. Pharmacol. Biochem. Behav..

[147] Hliňák Z., Krejčí I. (2002). N-Methyl-d-aspartate improved social recognition potency in rats. Neurosci. Lett..

[148] Takahashi A., Miczek K.A. (2014). Neurogenetics of aggressive behavior: Studies in rodents. Curr. Top. Behav. Neurosci..

[149] Newman E.L., Chu A., Bahamón B., Takahashi A., DeBold J.F., Miczek K.A. (2012). NMDA receptor antagonism: Escalation of aggressive behavior in alcohol-drinking mice. Psychopharmacology.

[150] Masugi-Tokita M., Flor P.J., Kawata M. (2016). Metabotropic glutamate receptor subtype 7 in the bed nucleus of the stria terminalis is essential for intermale aggression. Neuropsychopharmacology.

[151] Hildebrandt H., Dityatev A. (2015). Polysialic acid in brain development and synaptic plasticity. Top Curr. Chem..

[152] Bacq A., Astori S., Gebara E., Tang W., Silva B.A., Sanchez-Mut J., Grosse J., De Suduiraut I.G., Zanoletti O., MacLachlan C. (2018). Amygdala GluN2B-NMDAR dysfunction is critical in abnormal aggression of neurodevelopmental origin induced by St8sia2 deficiency. Mol. Psychiatry.

[153] McAllister K.H. (1994). D-Cycloserine enhances social behaviour in individually-housed mice in the resident-intruder test. Psychopharmacology.

[154] Covington H.E., Newman E.L., Tran S., Walton L., Hayek W., Leonard M.Z., DeBold J.F., Miczek K.A. (2018). The urge to fight: Persistent escalation by alcohol and role of NMDA receptors in mice. Front. Behav. Neurosci..

[155] Chang C.-H., Su C.-L., Gean P.-W. (2018). Mechanism underlying NMDA blockade-induced inhibition of aggression in post-weaning socially isolated mice. Neuropharmacol..

[156] Tóth M., Mikics E., Tulogdi A., Aliczki M., Haller J. (2011). Post-weaning social isolation induces abnormal forms of aggression in conjunction with increased glucocorticoid and autonomic stress responses. Horm. Behav..

[157] Chang C.H., Hsiao Y.H., Chen Y.W., Yu Y.J., Gean P.W. (2015). Social isolation-induced increase in NMDA receptors in the hippocampus exacerbates emotional dysregulation 1 in mice. Hippocampus.

[158] Zelikowsky M., Hui M., Karigo T., Choe A., Yang B., Blanco M.R., Beadle K., Gradinaru V., Deverman B.E., Anderson D.J. (2018). The Neuropeptide Tac2 Controls a Distributed Brain State Induced by Chronic Social Isolation Stress. Cell.

[159] Zhao X., Sun L., Jia H., Meng Q., Wu S., Li N., He S. (2009). Isolation rearing induces social and emotional function abnormalities and alters glutamate and neurodevelopment-related gene expression in rats. Prog. Neuro-Psychopharmacology Boil. Psychiatry.

[160] Sukhotina I.A., Bespalov A.Y. (2000). Effects of the NMDA receptor channel blockers memantine and MRZ 2/579 on morphine withdrawal-facilitated aggression in mice. Psychopharmacology.

[161] A Dravolina O., Belozertseva I.V., A Sukhotina I., Bespalov A.Y. (1999). Morphine tolerance and dependence in mice with history of repeated exposures to NMDA receptor channel blockers. Pharmacol. Biochem. Behav..

[162] Davis M.W., Khalsa J.H. (1971). Some determinants of aggressive behavior induced by morphine withdrawal. Psychon. Sci..

[163] Lal H., O’Brien J., Puri S.K. (1971). Morphine-withdrawal aggression: Sensitization by amphetamines. Psychopharmacology.

[164] Gellert V.F., Sparber S.B. (1979). Effects of morphine withdrawal on food competition hierarchies and fighting behavior in rats. Psychopharmacol.

[165] Tidey J.W., A Miczek K. (1992). Heightened aggressive behavior during morphine withdrawal: Effects of d-amphetamine. Psychopharmacology.

[166] Peregud D.I., Yakovlev A.A., Stepanichev M.Y., Onufriev M.V., Panchenko L.F., Gulyaeva N.V., Stepanichev M. (2012). Content of mRNA for NMDA Glutamate Receptor Subunits in the Frontal Cortex and Striatum of Rats after Morphine Withdrawal Is Related to the Degree of Abstinence. Bull. Exp. Boil. Med..

[167] Hull E.M., Dominguez J.M. (2007). Sexual behavior in male rodents. Horm. Behav..

[168] Dixon A.K., Huber C., A Lowe D. (1994). Clozapine promotes approach-oriented behavior in male mice. J. Clin. Psychiatry.

[169] Dominguez J.M., Balfour M.E., Lee H.S., Brown J.L., Davis B.A., Coolen L.M. (2007). Mating activates NMDA receptors in the medial preoptic area of male rats. Behav. Neurosci..

[170] Vigdorchik A.V., Parrish B.P., Lagoda G.A., McHenry J.A., Hull E.M. (2012). An NMDA antagonist in the MPOA impairs copulation and stimulus sensitization in male rats. Behav. Neurosci..

[171] Powell W.S., Dominguez J.M., Hull E.M. (2003). An NMDA antagonist impairs copulation and the experience-induced enhancement of male sexual behavior in the rat. Behav. Neurosci..

[172] Fleischmann A., Vincent P.A., Etgen A.M. (1991). Effects of non-competitive NMDA receptor antagonists on reproductive and motor behaviors in female rats. Brain Res..

[173] Hull E.M., Dominguez J.M. (2006). Getting his act together: Roles of glutamate, nitric oxide, and dopamine in the medial preoptic area. Brain Res..

[174] Kuroda K.O., Tachikawa K., Yoshida S., Tsuneoka Y., Numan M. (2011). Neuromolecular basis of parental behavior in laboratory mice and rats: With special emphasis on technical issues of using mouse genetics. Prog. Neuro-Psychopharmacology Boil. Psychiatry.

[175] Lonstein J.S., Gammie S.C. (2002). Sensory, hormonal, and neural control of maternal aggression in laboratory rodents. Neurosci. Biobehav. Rev..

[176] Sasaki K., Omotuyi O.I., Ueda M., Shinohara K., Ueda H. (2015). NMDA receptor agonists reverse impaired psychomotor and cognitive functions associated with hippocampal Hbegf-deficiency in mice. Mol. Brain.

[177] Niethammer M., Kim E., Sheng M. (1996). Interaction between the C terminus of NMDA receptor subunits and multiple members of the PSD-95 family of membrane-associated guanylate kinases. J. Neurosci..

[178] Muroi Y., Ishii T. (2019). Glutamatergic Signals in the Dorsal Raphe Nucleus Regulate Maternal Aggression and Care in an Opposing Manner in Mice. Neurosci..

[179] Muroi Y., Ishii T. (2015). Neuropeptide Y is crucial for nutritional state-dependent regulation of maternal behavior. Psychoneuroendocrinology..

[180] Da Veiga C.P., Miczek K.A., Lucion A.B., De Almeida R.M.M. (2010). Social instigation and aggression in postpartum female rats: Role of 5-Ht1A and 5-Ht1B receptors in the dorsal raphé nucleus and prefrontal cortex. Psychopharmacology.

[181] Malenfant S.A., O’Hearn S., Fleming A.S. (1991). MK801, an NMDA antagonist, blocks acquisition of a spatial task but does not block maternal experience effects. Physiol. Behav..

[182] Hol T., Berg C.V.D., Van Ree J., Spruijt B. (1999). Isolation during the play period in infancy decreases adult social interactions in rats. Behav. Brain Res..

[183] Hermes G., Li N., Duman C., Duman R. (2011). Post-weaning chronic social isolation produces profound behavioral dysregulation with decreases in prefrontal cortex synaptic-associated protein expressionin female rats. Physiol. Behav..

[184] Keesom S.M., Finton C.J., Sell G.L., Hurley L.M. (2017). Early-life social isolation influences mouse ultrasonic vocalizations during male-male social encoutners. PLoS ONE..

[185] Almeida-Santos A.F., Carvalho V.R., Jaimes L.F., De Castro C.M., Pinto H.P., Oliveira T.P.D., Vieira L.B., Moraes M.F.D., Pereira G.S. (2019). Social isolation impairs the persistence of social recognition memory by disturbing the glutamatergic tonus and the olfactory bulb-dorsal hippocampus coupling. Sci. Rep..

[186] Molenda-Figueira H.A., Bell M.R., De Lorme K.C., Sisk C.L. (2017). Pubertal pair-housing facilitates adult sexual behavior in male rats. Dev. Psychobiol..

[187] Kercmar J., Tobet S.A., Majdic G. (2014). Social isolation during puberty affects female sexual behavior in mice. Front. Behav. Neurosci..

[188] Jennings C.A., Robbins M.J., Cluderay J.E., Cilia J., Reid J.L., Taylor A., Jones D.N., Emson P.C., Southam E., Turnock-Jones J.J. (2009). Increased expression of the NR2A NMDA receptor subunit in the prefrontal cortex of rats reared in isolation. Synap.

